# Supplementary material for: Identifying and Overcoming Mechanisms of PARP Inhibitor Resistance in Homologous Recombination Repair-Deficient and Repair-Proficient High Grade Serous Ovarian Cancer Cells
Source: Cancers (Basel). 2020 Jun 9;12(6):1503. doi: 10.3390/cancers12061503 (PMC7353027; doi:10.3390/cancers12061503)
Supplement: Supplementary file 1 [file cancers-12-01503-s001.pdf]

Supplementary Materials

Identifying and Overcoming Mechanisms of PARP Inhibitor Resistance in Homologous Recombination Repair-Deficient and Repair-Proficient High Grade Serous Ovarian Cancer Cells

Miriam K. Gomez, Giuditta Illuzzi, Carlota Colomer, Michael Churchman, Robert L. Hollis, Mark J. O'Connor, Charlie Gourley, Elisabetta Leo and David W. Melton

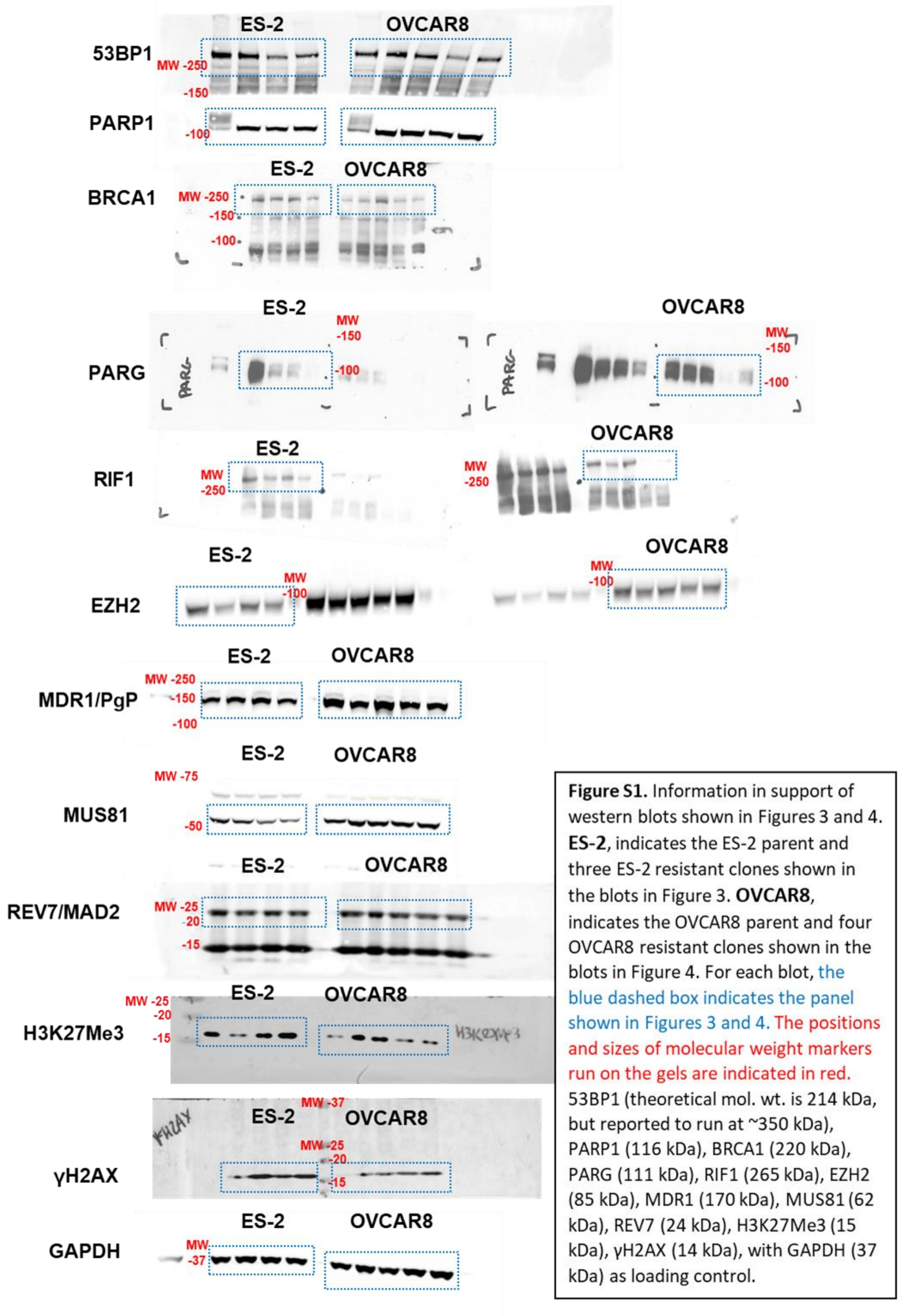

Figure S1. Information in support of western blots shown in Figures 3 and 4.

Table S1. Antibodies used for western blots.

Antibody	Supplier	Cat. No.	Dilution	Species
53BP1	Novus	NB100-305	1/1000	Rabbit
BRCA1 (Ab-1) (clone MS110)	Millipore	OP92	1/1000	Mouse mAB
EZH2	Cell Signaling	3147S	1/1000	Mouse
γH2AX (P-Ser139) (clone JBW301)	Merck Millipore	05-636	1/2000	Mouse mAB
H3K27Me3	Cell Signaling	9733S	1/1000	Rabbit
MDR1/PgP	Cell Signaling	12683	1/1000	Rabbit
Mus81	Sigma	M1445	1/2000	Mouse
PARG (clone D8B10)	Millipore	MABS61	1/1000	Mouse mAB
PARP1 (clone 46D11)	Cell Signaling	#9532	1/1000	Rabbit mAB
Rev7/MAD2	Abcam	ab180579	1/500	Rabbit
RIF1	Cell Signaling	95558-S	1/1000	Rabbit
GAPDH (clone 14C10)	Cell Signaling	#2118L	1/10000	Rabbit mAB

© 2020 by the authors. Licensee MDPI, Basel, Switzerland. This article is an open access article distributed under the terms and conditions of the Creative Commons Attribution (CC BY) license (http://creativecommons.org/licenses/by/4.0/).
